# Disseminated *Trichosporon asahii* infection in a combined liver‐kidney transplant recipient successfully treated with voriconazole

**DOI:** 10.1002/iid3.250

**Published:** 2019-04-20

**Authors:** Ranjit Sah, Arbindra singh Soin, Sony Chawla, Teena Wadhwa, Neha Gupta

**Affiliations:** ^1^ Department of Infectious Disease Medanta The Medicity Gurgaon Haryana India; ^2^ Institute of Medicine Tribhuvan University Teaching Hospital Kathmandu Nepal; ^3^ Institute of Liver Transplantation and regenerative Medicine Medanta The Medicity Gurgaon Haryana India; ^4^ Department of Microbiology Medanta The Medicity Gurgaon Haryana India

**Keywords:** combined liver‐kidney transplant, immunocompromised hosts, transplant recipient, *Trichosporon asahii*, voriconazole

## Abstract

**Introduction:**

*Trichosporon asahii* is an emerging cause of systemic fungal infection in an immunocompromised host. Several life threatening disseminated *T. asahii* infection in single solid organ (liver or kidney) transplant recipients, in neutropenic and hematological malignancy patients have been reported.

**Case presentation (Methods and Results):**

A 49‐year old gentleman who underwent simultaneous living‐donor liver transplantation (donor sister) and kidney transplant (donor wife) developed fever and subsegmental patchy consolidation with right sided pleural effusion on fourth postoperative day. Central line blood stream infection was suspected. Blood culture grew creamy white colonies of *T. asahii* on blood agar with characteristic dirty‐green colonies on CHROMagar. Laboratory analysis of pleural fluid also revealed budding yeast cells identified as *T. asahii*. Microscopy of the isolates showed hyphae, arthroconidia, and blastospores. The isolates were identified as *T. asahii* by VITEK MS which uses matrix‐assisted laser desorption/ionization time‐of‐flight (MALDI‐TOF) technology. Initially liposomal amphotericin B and micafungin was initiated, but due to lack of clinical and microbiological response, patient was switched to voriconazole. Simultaneously, tacrolimus doses were reduced to one‐third in view of interaction with voriconazole. Subsequently, patient improved with resolution of fever and microbiological cure.

**Conclusion:**

This is the first case report of disseminated *T. asahii* infection in a combined liver‐kidney transplant recipient successfully treated with voriconazole. Azole antifungal are the promising drug of choice for systemic *T. asahii* infection. Drug interactions should be considered while using these antifungal agents.

**Abbreviations**
*T. asahii*: *Trichosporon asahii*
MALDI‐TOF: matrix‐assisted laser desorption/ionization time‐of‐flight

## 1 BACKGROUND

*Trichosporon asahii* (*T. asahii*) formerly called *Trichosporon bigelli* is an emerging systemic fungal infection in immunocompromised hosts. It has been established as a cause of fatal life threatening invasive fungal infections (IFI) in patients with severe neutropenia, cancer, and hematopoietic stem cell transplant recipients. There are a few reports of *T. asahii* infection in patients with single solid organ transplant, human immunodeficiency virus infection, burns, catheter related infections, peritoneal dialysis, and prosthetic heart valves.^4^ Depending upon the immune status of the host, the clinical spectrum varies from localized skin lesions in immune‐competent host to disseminated systemic infections in immunocompromised patients.^5^ The majority of cases with invasive trichosporonosis have high (64%‐100%) mortality. Trichosporonosis may mimic candidiasis in both clinical presentation and histological appearance. Thus, a clear distinction in the mind of treating clinician is essential as the drug of choice is different for these IFI. An early diagnosis and initiation of an appropriate antifungal therapy can reduced the mortality. To the best of our knowledge, this is the first case of disseminated trichosporonosis in a combined liver and kidney transplant recipient who has been treated successfully.

## 2 CASE PRESENTATION

**Figure 1:**
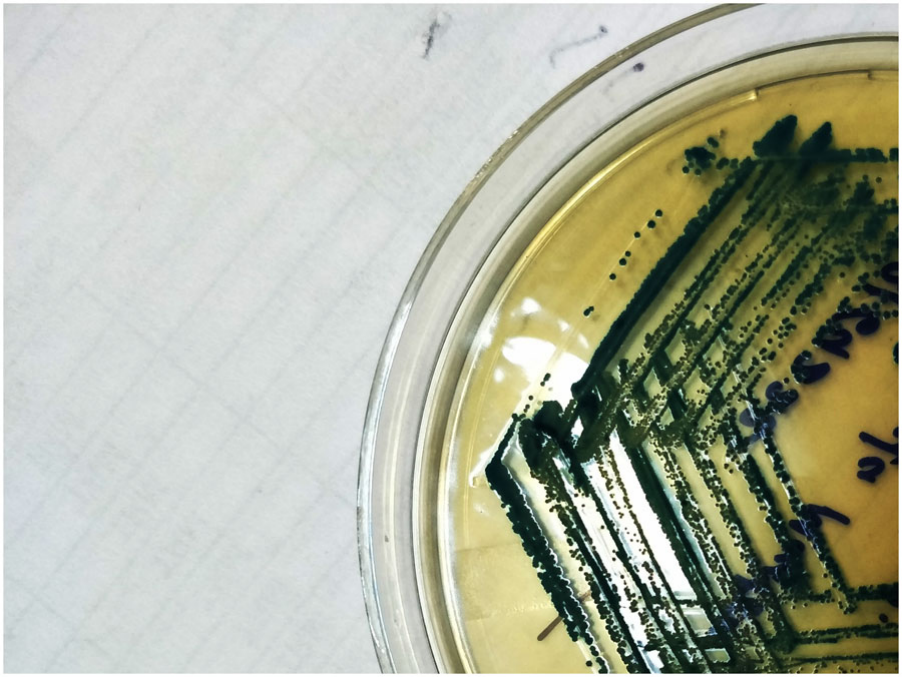
Characteristics dirty‐green colonies of *T. asahii* on CHROMagar

**Figure 2:**
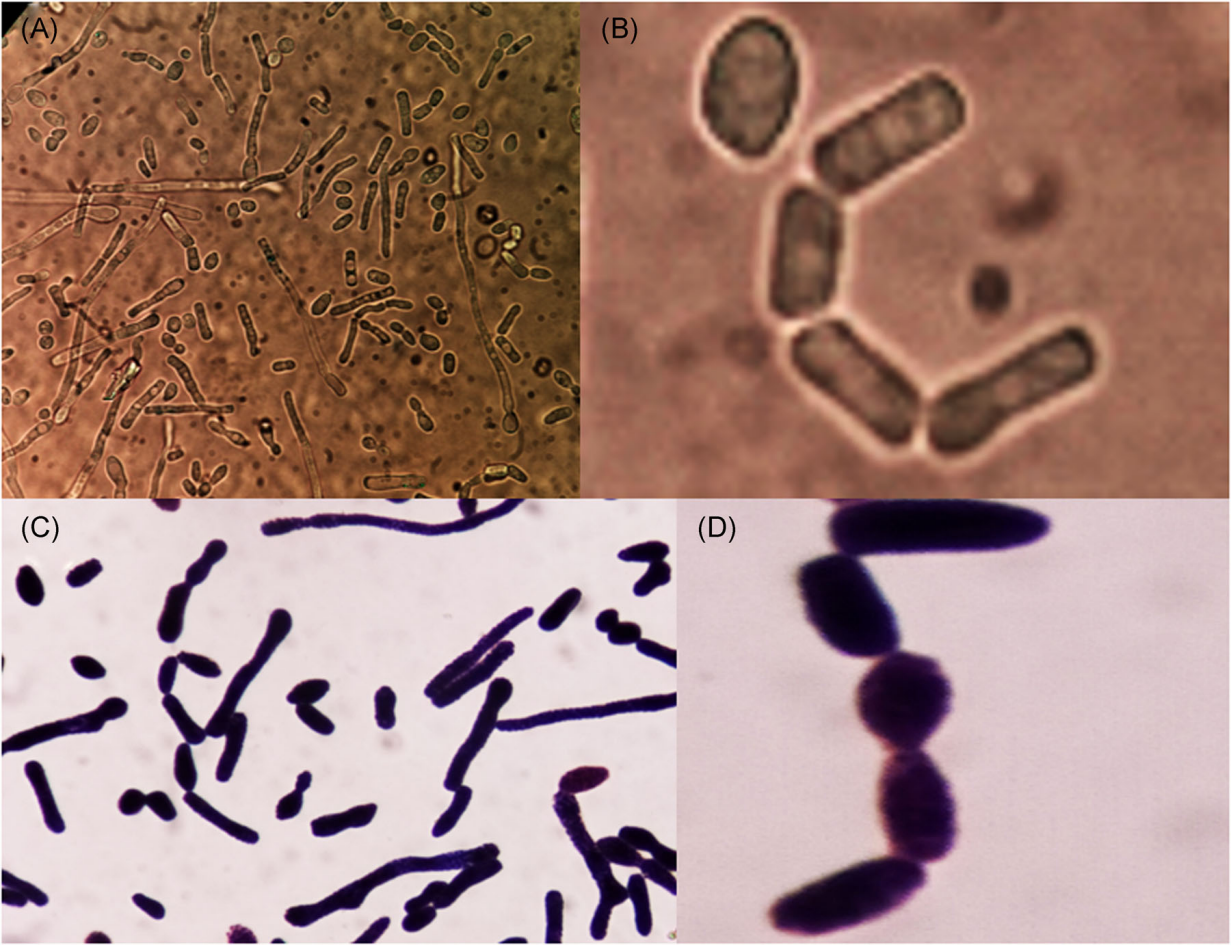
A, Hyphae, pseudohyphae, arthroconidia, and blastospores of *T. asahii* on wet mount preparation in x1000 magnification. B, Arthroconidia of *T. asahii* on wet mount preparation in x1000 magnification. C, Gram stain showing hyphae, pseudohyphae, arthroconidia and blastospores of *T. asahii* in x1000 magnification. D, Gram stain showing arthroconidia of *T. asahii* in x1000 magnification

A 49‐year old gentleman from Khania gaon, Assam, India with diabetes, hypertension with end‐stage renal disease and decompensated ethanol‐related chronic liver disease since 1 year underwent simultaneous living‐donor liver transplantation (donor sister) and kidney transplant (donor wife) in our institute. Recipient did not receive any induction therapy and was initiated on tacrolimus, mycophenolate mofetil, and wysolone. Four days post‐transplant, patient developed fever and subsegmental patchy consolidation with right sided pleural effusion. Central line blood stream infection was suspected. Blood culture were send which subsequently grew *T. asahii*. Laboratory analysis of pleural fluid also revealed budding yeast cells identified as *T. asahii*. Initially liposomal amphotericin B (L‐AmB; 3 mg/kg/day) was initiated but due to lack of clinical and microbiological response, micafungin (100 mg/day) was added to L‐AmB. His central venous catheter was changed. Because of the continuous fever on micafungin and persistent positive blood cultures with *T. asahii* an infectious diseases consultation was sought. On examination patient was having respiratory distress, laboratory investigation revealed leukocytosis (1 7007/µL) with neutrophilic (95.7%) predominance with normal creatinine and normal liver enzymes. Echocardiography did not revealed any vegetation. On examination of positive culture plates, there were creamy white colonies on blood agar and characteristic dirty‐green colonies on CHROMagar (Figure 1). Microscopy (wet mount, gram stain, and LPCB) revealed hyphae, arthroconidia, and blastospores (Figure 2A‐2D). The isolates were identified as *T. asahii* by VITEK MS which uses matrix‐assisted laser desorption/ionization time‐of‐flight (MALDI‐TOF) technology. *T. asahii* isolates were processed for drug susceptibility testing by broth microdilution according to *The Clinical and Laboratory Standards Institute (CLSI) Guideline* and were sensitive to fluconazole and voriconazole, but resistant to amphotericin B, flucytosine, and anidulafungin. On the basis of this, diagnosis of disseminated *T. asahii* infection in a combined living‐donor liver transplant and kidney transplant recipient was established. Hence, L‐AmB with micafungin was discontinued and patient was initiated on voriconazole. Tacrolimus doses were reduced to one‐third in view of interaction with voriconazole. The patient improved with resolution of fever. Repeat blood cultures on day 3 were negative. Voriconazole was prescribed for 14 days after the first negative blood culture. He was subsequently discharged and is doing well at 9 months of follow up.

### 2.1 Ethics approval

Patient gave written informed consent to publish this case report and related evidence.

### 2.2 Consent to publish

Local ethical guidelines state no need of ethical approval for a case report.

## 3 DISCUSSION AND CONCLUSION

*Trichosporon* species are found naturally in water, soil, and they may be a part of the normal human flora and colonize the skin, respiratory tract, gastrointestinal tracts, vagina, and urine.^9^ Of the 50 species of *Trichosporon* described so far, 16 have been pathogenic to human.^9^, ^10^ *T. asahii* has emerged as the leading cause of fatal opportunistic invasive deep seated infections followed by *T. mucoides* 11, 12 *T. ovoides, T. asteroides*, and *T. cutaneum* are associated with white piedra or other superficial infections. The sixth species, *T. inkin*, can cause superficial as well as disseminated infections.^11^ Trichosporonosis have a broad spectrum of clinical manifestation from self‐limiting cutaneous infections to fatal invasive infections which disseminated to different organ in the immunocompromised host. Disseminated infection with Invasive trichosporonosis is typically heralded by fever that is unresponsive to empiric antibacterial agents. Disseminated infections include fever, fungal sepsis, shock and patient may present with cutaneous lesions, pneumonia, chorioretinitis, and renal failure.^4^ In literature, it has been discussed that pneumonia is not a consistent or early feature, and thus portal of entry is not often apparent but in our patient right sided subsegmental patchy consolidation with pleural effusion was an early clinical manifestation. Skin lesions (papulonodular or pustular lesion that are sometimes necrotic) occur in 33% of patients.^15^ Skin biopsies of these lesions reveal *Trichosporon* species in more than three‐fourth of cases, and are useful in establishing an early diagnosis with prompt treatment.^6^ A higher mortality is likely with prolonged neutropenia, high burden of disease, a delayed time to diagnosis and with inappropriate antifungal therapy.^11^, ^15^

The diagnosis of trichosporonosis can be established by direct microscopic examination of the sample or by culture.^9^, ^12^ *T. asahii* is a urease‐positive, nonencapsulated yeast which can form both hyaline septate hyphae as well as pseudohyphae; and they produce cylinder‐shaped arthroconidia^9^, ^12^ these characteristic differentiate it from candida and other budding yeast like *Cryptococcus*, *Histoplasma capsulatam* . A urease‐positive yeast that forms arthroconidia can be presumptively identified as a *Trichosporon* species.^15^ Beside standard fungal media, *Trichosporon* species can be grown readily on routine bacterial culture media like on MacConkey agar and blood agar.^1^, ^15^ It shows white colonies with raised farinose surfaces on solid media, whereas it shows characteristic dirty‐green colonies on CHROMagar.^12^

In our case the *T. asahii* isolates were grown on blood agar. Culture characteristics resembles the *Candida* species but it does not form the germ tube as *Candida albicans*.^4^, ^17^ *Trichosporon* and other budding yeast like *Histoplasma*, *Cryptococcus*, and *Malassezia* species all are urease‐positive but *Trichosporon* species form arthroconidia, blastoconidia, hyphae & pseudohyphae whereas *Cryptococcus*, *Histoplasma*, and *Malassezia* do not.^15^ The isolates were subculture on CHROMagar which showed characteristics dirty‐green colonies.

The species of *Trichosporon* can be identified by standard laboratory procedures, such as morphological identification and the API 32C system (VITEK; bioMerieux, Marcy l'Etoile, France).^13^ Genetic confirmation can be done by MALDI‐TOF and sequencing of the ribosomal DNA IGS1 region, which is located between the 26S and 5S rRNA genes^13^ where IGS1 region is amplified by PCR.^13^ We have used MALDI‐TOF for genetic confirmation of our isolates.

Invasive trichosporonosis is difficult to treat and in vitro susceptibility assays to antifungal drugs have been standardized by broth microdilution technique as per *CLSI Guidelines*.^14^, ^18^ Poor response and failure of therapy with amphotericin B and echinocandins have been reported. The azole antifungals (fluconazole, voriconazole, posaconazole) have been shown to be effective against *T. asahii*.^7^ Of these, voriconazole can be preferably used.^14^, ^21^, ^22^ Newer antifungal isavuconazole also has activity against *T. asahii* and is a promising agent for these life threating infections. Drug interaction of these azole antifungal agents with tacrolimus should be considered. The dose of tacrolimus needs to be reduced by one‐third to two third, as voriconazole will increase the level or effect of tacrolimus by affecting hepatic/intestinal enzyme CYP3A4 metabolism. In literature 4 to 5 cases has been reported in liver transplant recipients with high mortality. Our patient was successfully treated with oral voriconazole. The patients who have survived from *T. asahii* infection were either not neutropenic or the neutropenia had recovered shortly after the infection was diagnosed.^15^ Early diagnosis, effective antifungal therapy, neutrophil count, the immune status, and the adjustment of dose of immunosuppressant with azoles play a crucial role in successful management of a patient with disseminated *T. asahii* infection.

## CONFLICT OF INTERESTS

The authors declare that there are no conflict of interests.

## AUTHOR CONTRIBUTIONS

RS, ASS, SC, TW, and NG established the diagnosis, managed the patient, designed the manuscript,and reviewed the literature and prepared the article for submission. All authors read and approved the final version of the manuscript.
